# Essential considerations in the investigation of associations between insulin and cancer risk using prescription databases

**DOI:** 10.3332/ecancer.2009.174

**Published:** 2009-12-11

**Authors:** P Boyle, I Ford, JFR Robertson, C La Vecchia, P Boffetta, P Autier

**Affiliations:** 1 International Prevention Research Institute, 95 cours Lafayette, 69006 Lyon, France; 2 Robertson Centre for Biostatistics, University of Glasgow, Glasgow, Scotland, UK; 3 Department of Surgery, University of Nottingham, Nottingham, UK; 4 Istituto ‘Mario Negri’, Milan, Italy

## Introduction

Studies of disease outcomes using administrative databases have identified a number of problems with the approach that suggest the need for a high level of expertise in their conduct. This is particularly true in the case of the investigation of the role of different insulins and the risk of cancer, which poses particular issues addressed below.

The greatest handicap is that the database has not been created specifically to study insulin as a cause of cancer, or indeed, cancer as an outcome. Given the relative rarity of some forms of cancer, it will frequently be necessary to perform simultaneous studies in different regions or countries to obtain enough events of interest to perform a meaningful study (in the statistical sense). This adds an extra degree of difficulty in that, since no two prescription databases are the same, these differences need to be taken into account at the start of the study.

All such observational studies must be conducted according to good research practice [1] and good epidemiology practice guidelines [2], including those specific to pharmaco-epidemiology [3]. However, there are a number of issues, some ***essential*** and some ***desirable***, specific to the study of insulin and cancer, which must be taken into consideration.

## Essential considerations for the study of insulin and cancer risk

Essential considerations for the study of insulin use and cancer risk fall under a number of broad headings: epidemiological methodology, diabetology, oncology and biostatistics.

Specific considerations in terms of the epidemiological methodology resolve around making sure that the study is designed to investigate the hypotheses of interest. Careful attention should be paid to issues surrounding confounding variables (***confounders***). These include body mass index (BMI), age, socio-economic status and duration and severity of diabetes: it would be very difficult to design a good study of insulin and cancer risk in the absence of this key information. It is also essential to declare the study hypotheses in advance so that the study can be designed and adequately powered (in terms of sample size) to investigate the hypotheses in a meaningful manner.

The study of diabetes, insulin and cancer is one in which the role of the diabetologist is crucial: it is not an area where epidemiologists and statisticians can work in isolation from the clinical situation. Most meaningful studies will involve conducting investigations, which should be as similar in design as possible, in different databases in different countries. Thus, it is critical to have information from a ***focus group*** of diabetologists and family doctors to obtain information regarding how the various therapies available are prescribed in the different health systems. It is critical to separate patients with type 1 diabetes from patients with type 2 diabetes in these epidemiological studies and to have complete coverage of a defined geographic population of all prescriptions issued. A complete history from the database of all prescriptions issued to the individual patient for their diabetes condition, including oral medications, is also essential.

The best approach, once all patients and their prescriptions have been identified, is to link this information with the Cancer Registry and the National Death Index (to obtain information on patients who have died). The Cancer Registry must be population-based and national to allow follow-up of patients who may have left the region covered by the prescription register. The Cancer Registry should be of good quality (specifically with a high level of population coverage and accuracy in terms of the diagnosis of cancers found) and cover at least the same geographic area as the prescription registry. An initial analysis must be run to exclude all patients from the study who have a prevalent cancer at the beginning of the defined period of the study.

Recognizing the complicated nature of such studies using prescription databases linked to cancer registries, it is vital to undertake careful and in-depth statistical analysis. The requirement for exploratory data analysis is overwhelming to allow the underlying structure of the dataset to become clear. Cox regression analyses with and without time-dependent covariates represent a sensible approach. Those who changed insulin during the study, ‘switchers’, and those who used more than one type of insulin simultaneously pose specific problems, which must be addressed. Restriction of the analysis to users of one type of insulin during the study period is inappropriate and likely to introduce bias. There are outstanding issues surrounding how to deal with the entire issue of ‘dose’, which require further exploration by the bio-statistical and epidemiological community.

## Conclusions

Analysis of associations between insulin and cancer risk, using prescription databases and cancer registries is complicated and should only be undertaken with extreme caution. However, even though these prescription databases were not established with such analyses in mind, it is possible to obtain clear information from them if (and only if) the study is designed adequately, key data are available and there is good bio-statistical and epidemiological capability available to analyse and interpret the data in a satisfactory manner.

In Table 1, the essential elements for conducting a meaningful study of insulin use and cancer risk, using administrative databases, are presented. When evaluating a publication on this topic, if the check list results in a number of ‘no’ responses then the value of the study is dubious and the interpretation of the findings must be viewed with great caution.

## Figures and Tables

**Table 1:**
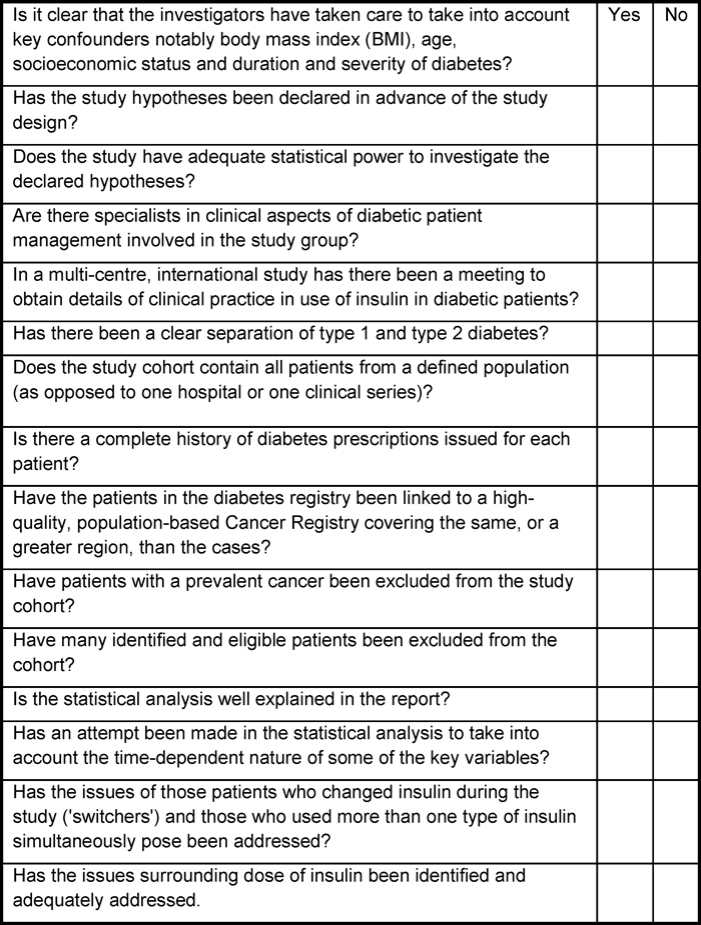
Essential check list or evaluation of quality for studies of insulin and cancer risk using prescription databases
